# Fusing NIR and Process Sensors Data for Polymer Production Monitoring

**DOI:** 10.3389/fchem.2021.748723

**Published:** 2021-10-22

**Authors:** Lorenzo Strani, Erik Mantovani, Francesco Bonacini, Federico Marini, Marina Cocchi

**Affiliations:** ^1^ Department of Chemical and Geological Sciences, University of Modena and Reggio Emilia, Modena, Italy; ^2^ Research Center, Versalis (ENI) S.p.A., Mantvoa, Italy; ^3^ Department of Chemistry, University of Roma La Sapienza, Roma, Italy

**Keywords:** process monitoring, data fusion, MSPC charts, NIR online, styrenic polymers

## Abstract

Process analytical technology and multivariate process monitoring are nowadays the most effective approaches to achieve real-time quality monitoring/control in production. However, their use is not yet a common practice, and industries benefit much less than they could from the outcome of the hundreds of sensors that constantly monitor production in industrial plants. The huge amount of sensor data collected are still mostly used to produce univariate control charts, monitoring one compartment at a time, and the product quality variables are generally used to monitor production, despite their low frequency (offline measurements at analytical laboratory), which is not suitable for real-time monitoring. On the contrary, it would be extremely advantageous to benefit from predictive models that, based on online sensors, will be able to return quality parameters in real time. As a matter of fact, the plant setup influences the product quality, and process sensors (flow meters, thermocouples, etc.) implicitly register process variability, correlation trends, drift, etc. When the available spectroscopic sensors, reflecting chemical composition and structure, consent to monitor the intermediate products, coupling process, and spectroscopic sensor and extracting/fusing information by multivariate analysis from this data would enhance the evaluation of the produced material features allowing production quality to be estimated at a very early stage. The present work, at a pilot plant scale, applied multivariate statistical process control (MSPC) charts, obtained by data fusion of process sensor data and near-infrared (NIR) probes, on a continuous styrene-acrylonitrile (SAN) production process. Furthermore, PLS regression was used for real-time prediction of the Melt Flow Index and percentage of bounded acrylonitrile (%AN). The results show that the MSPC model was able to detect deviations from normal operative conditions, indicating the variables responsible for the deviation, be they spectral or process. Moreover, predictive regression models obtained using the fused data showed better results than models computed using single datasets in terms of both errors of prediction and *R* ^2^. Thus, the fusion of spectra and process data improved the real-time monitoring, allowing an easier visualization of the process ongoing, a faster understanding of possible faults, and real-time assessment of the final product quality.

## Introduction

A large number of sensors, such as thermocouples, pressure gauges, and flow indicators, which generate an enormous amount of data, are normally installed in petrochemical production plants. The plant operators use these process sensors to control production and monitor operating conditions (Kourti and MacGregor, 1995). The aim is to reduce production faults and defects resulting from accidental plant malfunctions, changes in product characteristics (molecular weight, particle size, etc.), and nonoptimal conditions, caused by the complexity of the process or by its tendency to get contaminated that generates frequent maintenance needs. The collected data are used for the control and optimization of processes and also for extracting significant information to predict the properties that define the quality of the final product in real time. Furthermore, in all industrial processes, energy saving, efficient use of raw materials, and optimal production planning are essential. The measurements made by the sensors in the plants can be used for these needs. The production control in the petrochemical industry, as well as in many others, is based on the knowledge and experience of the technical operators and is mainly supported by single univariate control charts developed for a few selected sensors and monitoring points (Chaudhry and Higbie, 1989). The control is carried out by verifying that the values of the selected parameters fall within a predetermined and carefully chosen confidence interval. As a process always presents variability, it is fundamental to define the standard operating conditions, according to which the process can be considered stable around its natural variability and therefore within the confidence limits of the monitored process parameters (Ferrer-Riquelme, 2009). The plant operators are perfectly aware of the optimal values of the parameters and their confidence intervals, but since more than one variable is used to monitor the entire process, it results in a large number of control charts to pay attention to. When the process encounters an anomaly and goes out of the range of standard operating conditions, it is very likely that several parameters would change simultaneously, due to the correlation that exists between the variables, and it would be very difficult for operators to identify the source of the problem. The sources of variability during production can be related to impurities, defective sensors, plant aging, leaks, and many other possible causes.

Multivariate statistical process control, instead of focusing on individual variables, focuses on the entire group of process variables and their correlation (Kourti, 2006). In this way, the plant operators can identify anomalies, reset the plant parameters, change the raw material, and, in general, properly fix all the other possible events that cause a change in the conditions of the process. This method allows for monitoring the production through few multivariate control charts. It is based on the concept of benefiting from the correlation structure of the process variables, which allows the compression of the responses of a large number of sensors into a few components (the latent variables). In this way, it will be possible to parsimoniously describe the sources of variability in the process (Kourti, 2009) and its time evolution by a few selected trajectories and establishing confidence limits in order to show how far the current condition is from the desired or normal operating situation.

Process sensors that typically measure temperature, pressure, flow, etc., provide information of the process ongoing, but they do not allow the operators to directly know the status of the product. In order to obtain chemical and physical information of the product in real time, near-infrared (NIR) spectroscopic probes are often installed in crucial steps of the process. NIR spectroscopy performs fast, and it is nondestructive and low-invasive on/inline measurements, making it perfectly suitable for being used as a process analyzer. The fusion of NIR data with process sensors data to build multivariate statistical process control (MSPC) charts provided successful results in the three different examples proposed by de Oliveira et al., 2020, in the pharmaceutical and petrochemical fields. In general, some studies conducted in collaboration with petrochemical companies reported the use of multivariate statistical control methods, showing numerous successes (Skagerberg et al., 1992; Macho and Larrechi, 2002; Kourti, 2005; Ferrer, 2007; Bonacini et al., 2013; de Oliveira et al., 2017), suggesting that in recent years, industries have opened up to the use of multivariate techniques, taking advantage of them.

In this context, the present work aimed at building PCA-based MSPC charts from the data fusion of spectroscopic data collected by two NIR probes (located at an early reaction step and close to the final stage, respectively) with process sensors data on a continuous styrenic polymers production process. Furthermore, PLS regression was used for the real-time prediction of selected quality parameters.

## Materials and Methods

### Plant Description

**FIGURE 1:**
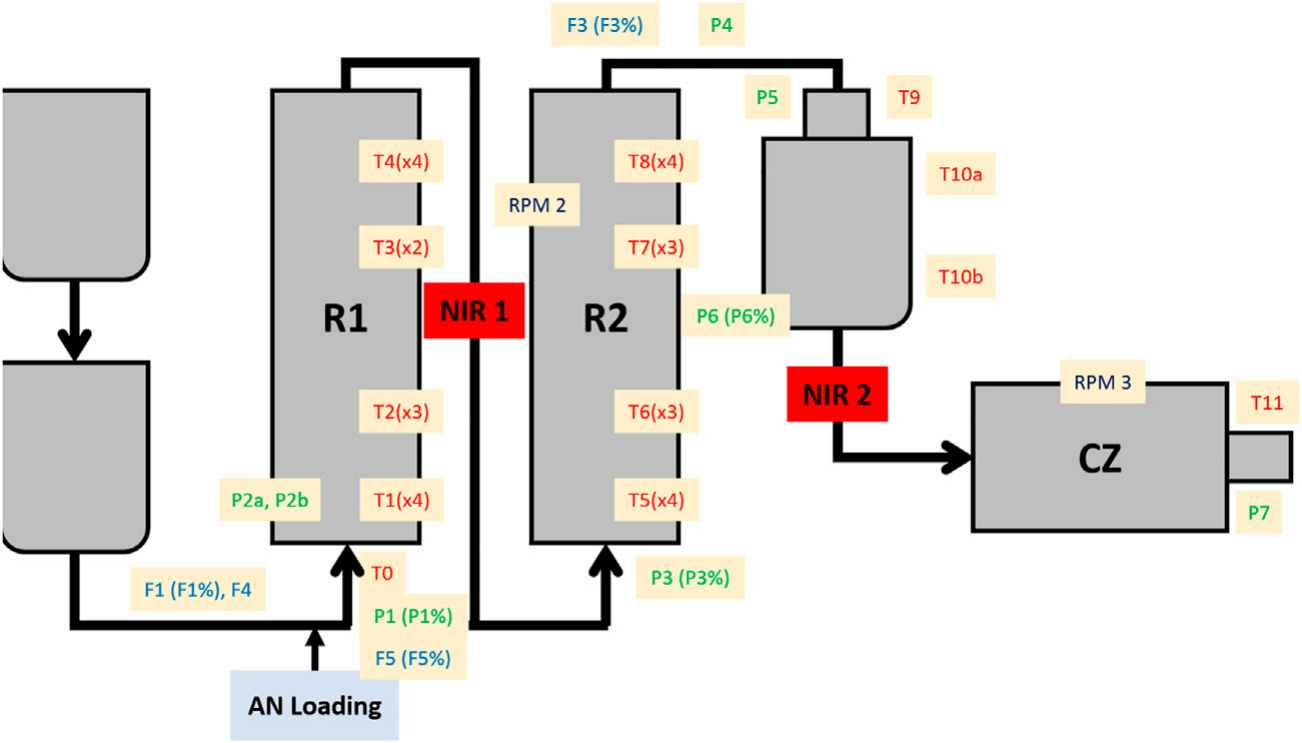
Schematic representation of the SAN production plant. R1 = first reactor; R2 = second reactor; NIR1 = first NIR probe; NIR2 = second NIR probe; CZ = cutting zone; AN = acrylonitrile; T = temperature sensors; P = pressure sensors; F = flow sensors; RPM = motor speed sensors. A percentage symbol after the sensor name indicates the opening extent of a valve linked to the specific sensor.

The monitoring of the styreneacrylonitrile (SAN) production has been carried out in the Versalis (ENI) company industrial pilot plant, operating continuously. A schematic representation of the plant is shown in Figure 1. The most relevant plant sectors for the present study are the two reactors (R1 and R2), where the polymer formation occurs, and the cutting zone (CZ), a final section where the finished product, i.e., the polymer, is reduced by cutting in small pieces. A total of 52 process sensors are installed throughout the process lines, of which 32 are for measuring the temperature, 11 for the pressure, 7 for the flow, and 2 for the motor speed. Furthermore, two NIR probes were installed in crucial steps of the process: one between R1 and R2 (NIR1) and the other right before the CZ (NIR2).

The monitoring of the SAN production occurred from February 4 to February 23, 2016, and the data were collected every 5 min. In this period, there was a deliberate variation of settings for some of the process sensors at the end of February 11, a pause and restart of the production during the morning on February 12, and a change in the formulation of the product on February 15 (i.e., an increase of the chain transfer amount). The settings variation was carried out in order to test how the plant would react to this kind of modification in view of the chain transfer amount increase.

### Reference Analysis

With the aim of assessing the quality of SAN polymer, two different parameters were evaluated: Melt Flow Index (MFI) and percentage of bound acrylonitrile. SAN samples were immediately collected after being cut and brought to the laboratory for the offline analyses.

MFI is an analysis that indicates the fluidity of a molten polymer, providing information about the fluid dynamic behavior of the material. The analysis is carried out by measuring the quantity of matter in grams that passes through a capillary (with a known and standard section) at a temperature of 220°C under the pressure of a weight of 10 kg in 10 min. The results generally range from 4 g, which denotes a very hard product, to 30 g, indicating a highly fluid product, and depend on the molecular weight and on the possible presence of fluidifying agents (Shenoy and Saini, 1986). In this study, 196 MFI analyses were carried out, ranging from 3.1 to 18 g and covering homogeneously the considered time range.

The amount of bonded acrylonitrile (%AN) in SAN samples is measured in order to define how much chemical and thermal resistance the material has. To determine %AN amount in the SAN copolymer, an NIR analysis is performed offline with a Matrix FT-NIR spectrometer (Bruker Optics, Milan, Italy). The sample, in the form of a granule, is analyzed with an integrating sphere, and two NIR spectra are recorded for each of them. A Vario El Elementar (Waltham, MA, United States) CHNS elemental analyzer, used as a reference method, calibrated the NIR spectrometer (the multivariate calibration curve was previously established by PLS regression). In total, 218 %AN analyses were performed ranging from 13.37 to 16.6%, covering homogeneously the considered time range.

### NIR Spectroscopy

The on-line monitoring of SAN production was carried out with a Matrix FT-NIR spectrometer (Bruker Optics, Milan, Italy), connected with a probe (HT immersion probe, Drawing-no. 661.2350_1, Hellma GmbH and Co. KG, Müllheim, Germany) via optical fibers (length: 50 m, diameter: 600 μm). These special polymer fibers are directly coupled to the process pipe in high temperature and stress conditions. Spectra were collected in transmission mode (path length: 5 mm) every 5 min in the whole NIR spectral range (12,500–4,000 cm^−1^) for a total of 5,434 acquisitions, with a resolution of 4 cm^−1^ and 64 scans for both background and spectra.

### Data Analysis

#### Datasets

**FIGURE 2:**
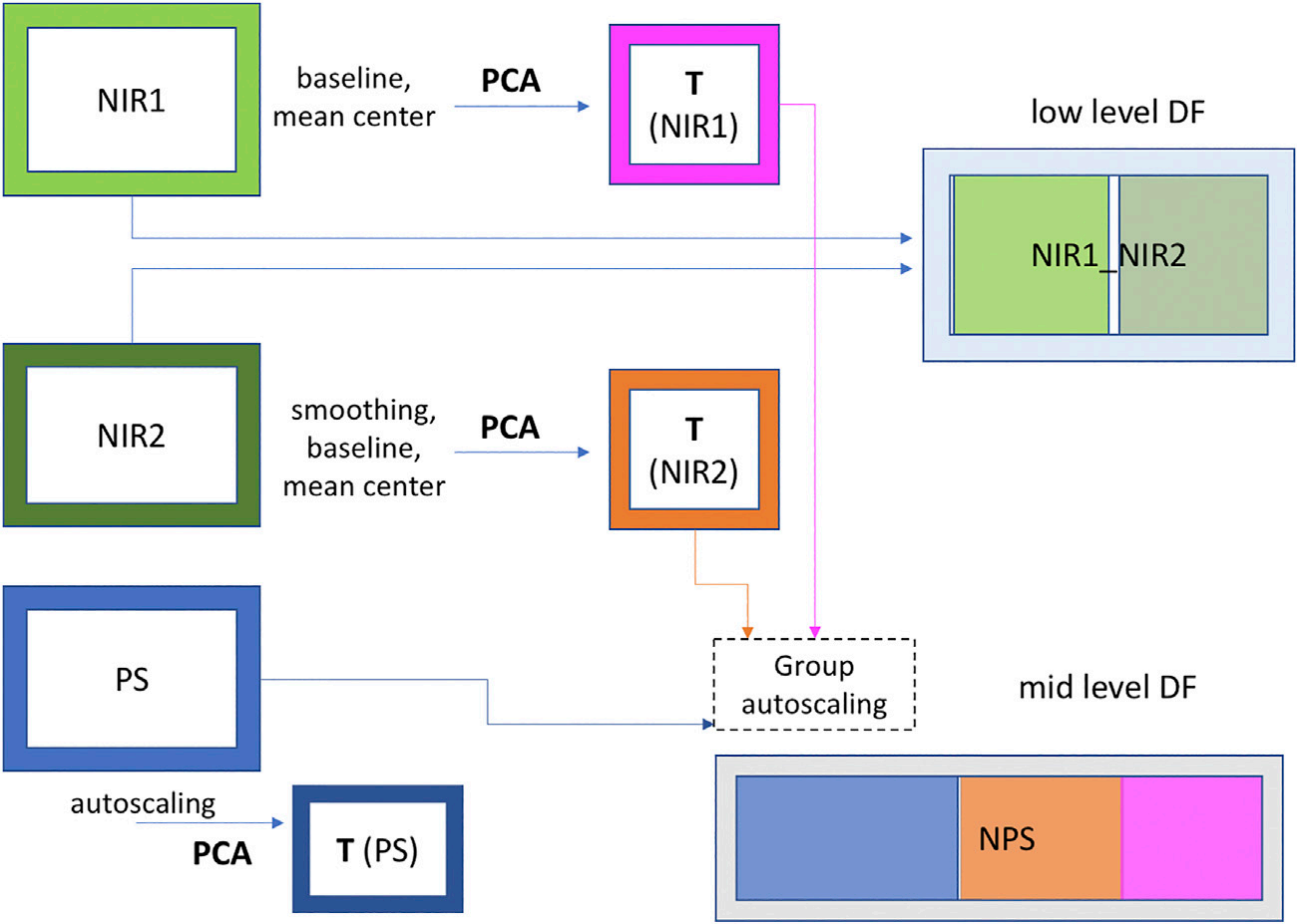
Schematic representation of data sets assembly.

Data were arranged in three different datasets: two containing the spectra collected by NIR1 and NIR2, and a third one containing the process sensor data (PS). These datasets were analyzed both singularly and merged together, applying low- and mid-level data fusion techniques. A schematic representation of data arrangement is shown in Figure 2. The low-level data fusion was achieved simply concatenating NIR1 and NIR2 datasets row-wise, obtaining a single dataset with the same number of rows (data points) as the previous ones, but with twice the number of columns (wavenumbers). For the mid-level data fusion, two steps were required: first, the information contained in NIR1 and NIR2 datasets was extracted via PCA, selecting the proper number of PCs with the aim of retaining just the relevant information contained in the data. Then, the features (scores) obtained in this way were concatenated with the PS dataset, creating a single dataset containing the information of both NIR probes and process sensors data (NPS). The datasets assembly was performed taking into account the residence time according to the position of each sensor and NIR probe along the process line and the process itself. In this way, each data point present in the datasets, which contains information collected at different times, was referred to the same material.

The spectral range considered for the data analysis was 6,200–4,700 cm^−1^, as other regions were characterized by high noise and baseline regions, i.e., no bands linked to either reactant or product are present. Spectra were pretreated in order to improve the quality of the analysis. In particular, automatic weighted least square method has been used for the baseline correction, followed by mean centering. Furthermore, only for the spectra acquired by NIR 2, prior to baseline correction, smoothing (SavitzkyGolay method, filter width seven points, polynomial order 1) was applied with the purpose of reducing noise. Autoscaling followed by block scaling was applied on the NPS dataset in order to avoid that a single block of data (NIR1 and NIR2 features and PS) could contribute more than the others just for containing a greater number of variables.

#### PCA and MSPC Charts

PCA, described by Eq. 1, was used both to perform the initial exploratory data analysis and to build MSPC charts.

$$X=TP^{T}+E.$$ (1)

Here, **X** is a data matrix composed of *m* rows (samples) and *n* columns (variables). The scores matrix **T** describes how each sample relates to each other, whereas the loading matrix **P** contains information about the influence of the measured variables on the model and their correlation structure. **E** is the residual matrix, which contains the unmodeled variation, has the same dimensions of **X**, and it is obtained by subtraction of the reconstructed (by the PCA model) data $\left(TP^{T}\right)\;$ from **X**. Thus, the original data is compressed into a fewer number of independent variables, i.e., principal components (PCs), orthogonal to each other. Therefore, a new projection space is created, smaller in size, whose coordinates are represented by the PCs.

The PCA-based MSPC chart models were built using the data from February 4 to February 10, before the variation of some of the process settings, whereas data from February 11 to February 15 were used to validate the model. Data points acquired after February 15, corresponding to the formulation change, were not used in this part of the work. The cross-validation scheme used for the internal validation of the models was contiguous blocks with ten cancelation groups, in order to mimic the routine situation in which the monitoring MSPC model is going to be applied.

MSPC is based on two distinct monitoring charts reporting as function of time the distance in PCA scores space (T^2^) and the squared residuals (Q), respectively:

$$T_{i}^{2}=\sum_{a=1}^{A}\frac{t_{ia}^{2}}{\lambda_{a}}$$ (2)

$$Q_{i}=\sum_{m=1}^{M}e_{im}^{2}$$ (3)

where *t*_*ia*_ is the score value for the *ath* component of a given sample (time point observation *i*), *λ*_*a*_ the corresponding eigenvalue, and *e*_*im*_ its residual value for a given variable *m*.

The T^2^ and Q acceptance limits are calculated based on Hotelling-T^2^ (Nomikos and Mac Gregor 1995) and χ^2^ statistics, calculated with the Jackson and Mudholkar approximation, respectively.

The T^2^ parameter indicates the distance of a sample in the model space, which means that a sample with a high T^2^ value has a distance from the center of the model larger than what is usually expected but is still described properly by the model. On the other hand, the Q parameter describes the distance of a sample from the model space, indicating an anomalous condition with respect to the optimal operating conditions, i.e., the conditions under which the model was built.

Once an anomalous sample is detected to assess the sensors responsible for the deviation, the T^2^ or Q contribution plots, depending on which chart detected it, can be displayed. The contributions to T^2^ for the *i*th sample, ***t***_*con,i*_, are a vector calculated from:

$$t_{con,i}=t_{i}\lambda^{-1/2}p^{T}.$$ (4)

Here, **P** is the loading matrix (n° of variables x n° of components) and $P^{T}$ its transpose.

While the **Q**_*i*_ contribution is simply a vector holding the *i*th sample squared residuals for each sensor multiplied by its sign, the contribution plots can aid fault diagnosis. In a T^2^ contribution, a high absolute value of the contribution of a given variable denotes a problem with that specific variable which assumes an extreme value, higher or lower depending on the sign of the contribution, with respect to the other ones. The interpretation of Q contribution is less straightforward because it signals that the correlation structure of the variables (with a high absolute value of the contribution) has changed. Thus, if, e.g., two variables have a high positive and negative contribution value, respectively, it could be that for the corresponding out-of-control observations, these variables are inversely correlated, while for the normal operative conditions observation, they were directly correlated. Inspection of scatter plot of one variable vs. the other may be used to have a confirmation (Westerhuis et al., 2000).

#### Predictive Models

PLS regression was used with the aim of developing predictive models of SAN quality in real time. Venetian blinds cross-validation with ten cancelation groups was used to establish the number of PLS components. The external validation of the PLS models was performed using a test set whose sample was not used for the model computation. Since MFI and %AN reference analyses were not always performed on the same samples and the number of the two kinds of analyses was not the same, PLS models and predictions were carried out as follows: the models were calculated using the 130 samples on which both analyses were made, whereas the predictions were performed using samples on which only one of the two determinations was carried out, i.e., 66 for MFI and 88 for %AN. To evaluate the reliability of the models both RMSECV and RMSEP, i.e., the root mean square error in cross-validation and in prediction, respectively, and the corresponding values of the coefficient of determination (R2) were taken into account. A total of 4 PLS models were computed, three using as X block each of the three datasets NIR1, NIR2, and PS individually, and the last one using the fused NPS dataset. Besides, the Y block contains the results of the MFI and %AN analysis together (PLS2 models); even if, for the reasons mentioned above, predictions on the validation samples were evaluated separately. Autoscaling was applied on Y block, since it contains values obtained with different techniques, having different ranges and scales.

#### Software

Data elaboration has been carried out by using PLS Toolbox (version 8.9, Eigenvector Research Inc. WA, United States) (MathWorks, MA, United States).

## Results and Discussion

### Exploratory Data Analysis

**FIGURE 3:**
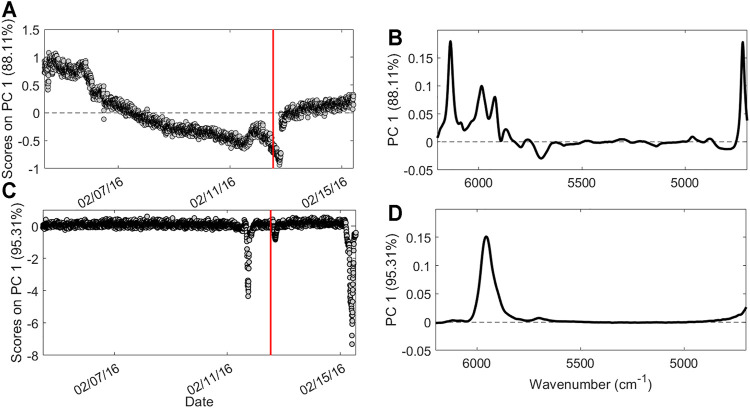
Results of the Exploratory Data Analysis performed on spectral data. Scores as a function of time **(A)** and loadings **(B)** on PC1 for NIR1 dataset; scores as a function of time **(C)** and loadings **(D)** on PC1 for NIR2 dataset. Red bar indicates the moment of the production pause.

**FIGURE 4:**
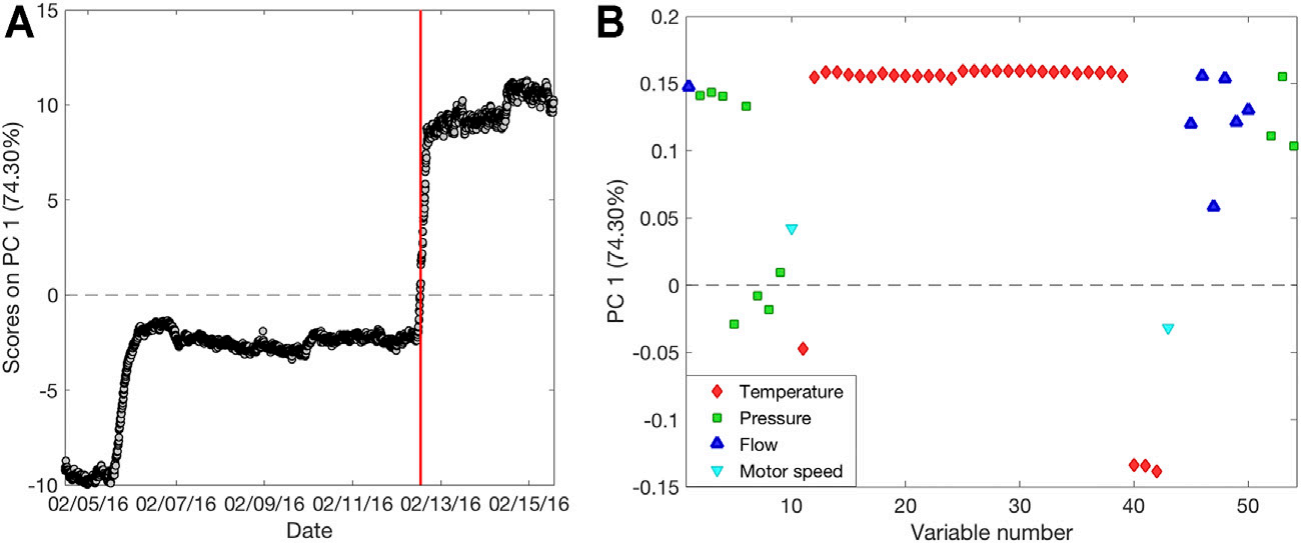
Results of the Exploratory Data Analysis performed on process sensors data. Scores as a function of time **(A)** and loadings **(B)** on PC1 for PS dataset. Red bar indicates the moment of the production pause.

Each different data block was analyzed with PCA in order to visualize and extract features and relevant information on the process. The first PCA analysis was carried out on the NIR1 dataset, choosing five PCs for the model computation that explain 95% of the total variance. Figure 3A represents the scores on the first PC as a function of time. It is possible to observe a slow but constant decrease of the scores over time, until the temporary stop of the production, highlighted by the red bar. During the last 2 days of production, samples start to increase their score values, behaving differently from the previous ones. The spectral bands responsible for the data variation are shown in the loadings plot (Figure 3B). Bands at 6,130, 6,000, and 4,720 cm^−1^ can be ascribed to the styrene monomer, whereas the band at 5,900 cm^−1^ is related to the forming SAN polymer (Takeuchi et al., 1968). These bands present higher intensity in samples with positive scores and lower intensity in samples with negative scores, suggesting a slow decrease overtime of their intensity until the production stops. Figure 3C shows the scores of the first PC as a function of time related to the PCA performed on the NIR2 dataset. Also, in this case, five PCs were selected for the model computation, explaining 99.8% of the total variance. At this final stage, a general more stable trend over time is observed, with the exception of three distinct moments: 20 h before and 4 h after the production stops, and at the very end of the period taken into account. Looking at the corresponding loadings plot (Figure 3D), it can be observed how these extreme samples have negative scores, meaning that with respect to the other time points, they are characterized by a less intense band at 5,900 cm^−1^, suggesting a lower extent of polymer formation. Finally, PCA was also carried out on the PS dataset (Figure 4). In this respect, the model was computed considering three PCs explaining 84.9% of the total variance. The scores of PC1 as a function of time (Figure 4A) provide a different trend than those of the PCA performed on spectral data, as in this case, the measurements made after the production stop, with positive scores, resulted clearly different from the others without returning to the stable range of values before the stopping. The loadings plot (Figure 4B) explains how samples collected after the production pause show, among others, high values for temperature sensors linked to the two reactors (T1–T8).

### MSPC Charts

From these PCA models, it is clear how each data block provides different information about the processes; therefore, two different data fusion approaches were applied. The low-level data fusion approach was performed merging NIR1 and NIR2 datasets, in order to gather the spectral information collected in the two key steps of the process, namely between the two reactors and before the CZ. The PCA carried out on this dataset (data not shown for the sake of brevity) confirmed what already showed by PCA performed on NIR1 and NIR2 datasets separately. Scores and loadings profiles related to PC1 and PC2 are almost identical to the ones obtained by NIR2 and NIR1 PCA models, respectively. Furthermore, MSPC charts based on T^2^ and Q were built with the modality described in chapter 2.4.2. The results obtained were good, but it would be difficult for the plant operators to understand the nature of an occurring problem, being the spectral interpretation above their expertise. For this reason, a mid-level data fusion approach was applied, considering also the information contained in the process sensors data, i.e., PS dataset. Hence, NPS dataset was created merging the scores obtained from PCA performed on NIR1 and NIR2 datasets together with PS data. A further PCA was carried out, using three PCs to build the model. Also, in this case, MSPC charts were computed as described in chapter 2.4.2.

**FIGURE 5:**
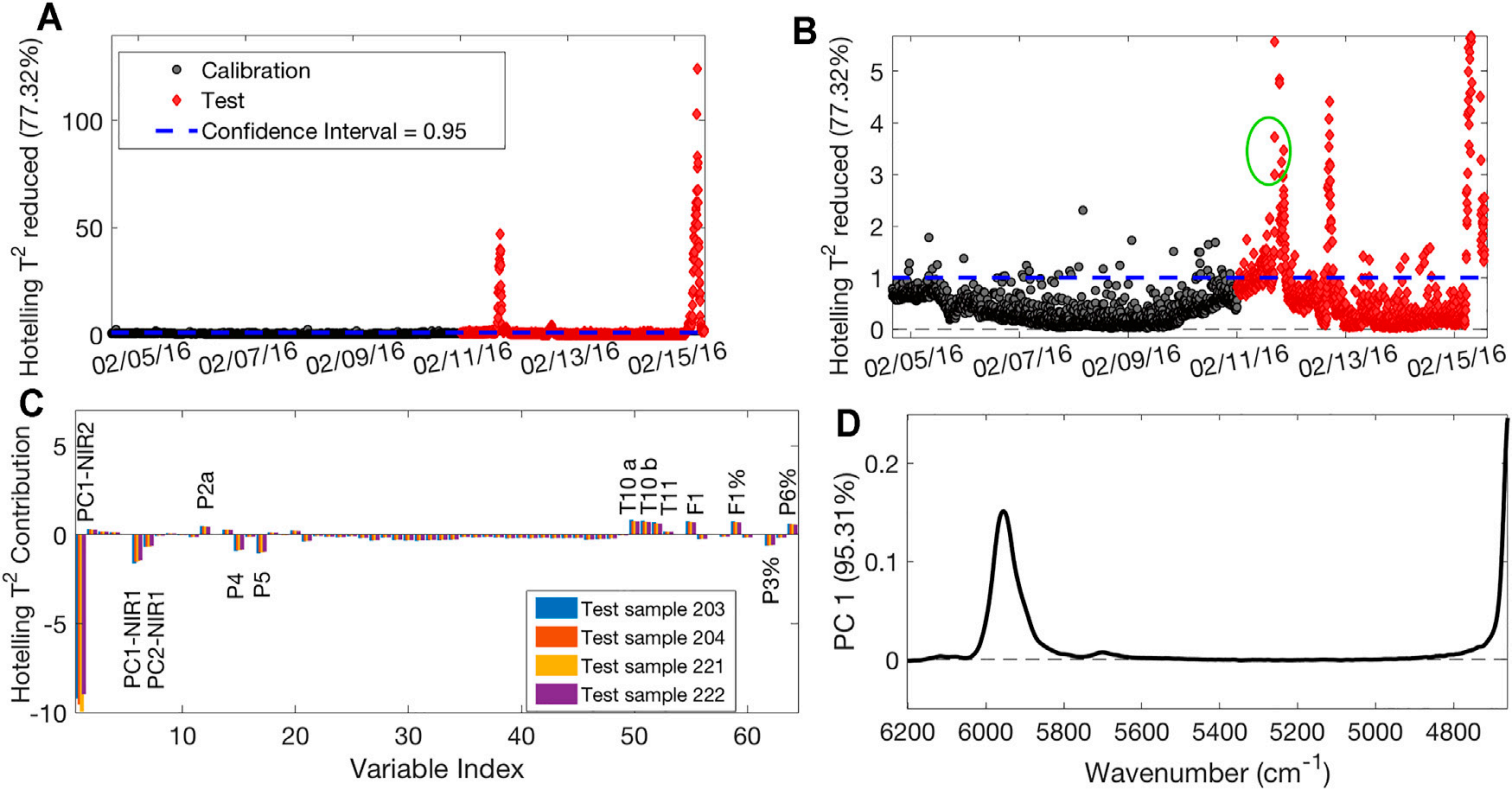
T^2^-based MSPC chart **(A)**; zoom on the confidence limit area **(B)**; contribution plot of the four circled samples of **Panel B (C)**; loadings plot on PC1 of PCA performed on NIR2 dataset **(D)**.

Figure 5A shows the MSPC chart related to the T^2^ parameter, which describes the distance of each sample from the origin within the model space. Figure 5B is a zoom of Figure 5A close to the confidence interval area. Black circles represent the calibration samples used to build the model, as they can efficiently represent optimal operative conditions according to plant experts, whereas red diamonds indicate the validation samples projected on the model. The calibration samples are almost all inside the 95% confidence interval, with some isolated exceptions of samples falling just outside the interval. Since neither consecutive set of calibration samples outside the confidence interval nor samples falling too far away from it were present, these isolated samples were kept in the model. There are three different clusters of validation samples that are outside the confidence interval: a first group corresponds to time observations taken 20 h before the production stop, and a second group corresponds to time observations taken 4 h after it, as described by the first PC of NIR2 PCA. The third group is observed at the end of the monitored time. The T^2^ contribution plot of the samples of the first two clusters reveals that the PC1 scores linked to the second NIR probe are the variable mostly responsible for this behavior. As an example, the contribution plot of the four circled samples in Figure 5B is shown in Figure 5C. Since the loadings related to this PC can be ascribed to the SAN band at 5,900 cm^−1^ (Figure 5D), it follows that the anomalous samples present a lower polymer conversion. However, also PC1 scores related to the first NIR probe were found relevant to explain this difference, proving that a probe that collects spectra of an intermediate product is useful, as it could provide information on possible faults well in advance with respect to the second one. Furthermore, it is possible to detect the process sensors linked to the sample’s abnormality which can suggest possible reasons for the deviations. In this case, among others, sensors T10a, T10b, and T11 in the zone between R2 and CZ registered values higher than the ones registered for calibration samples, indicating a possible problem in that specific zone.

**FIGURE 6:**
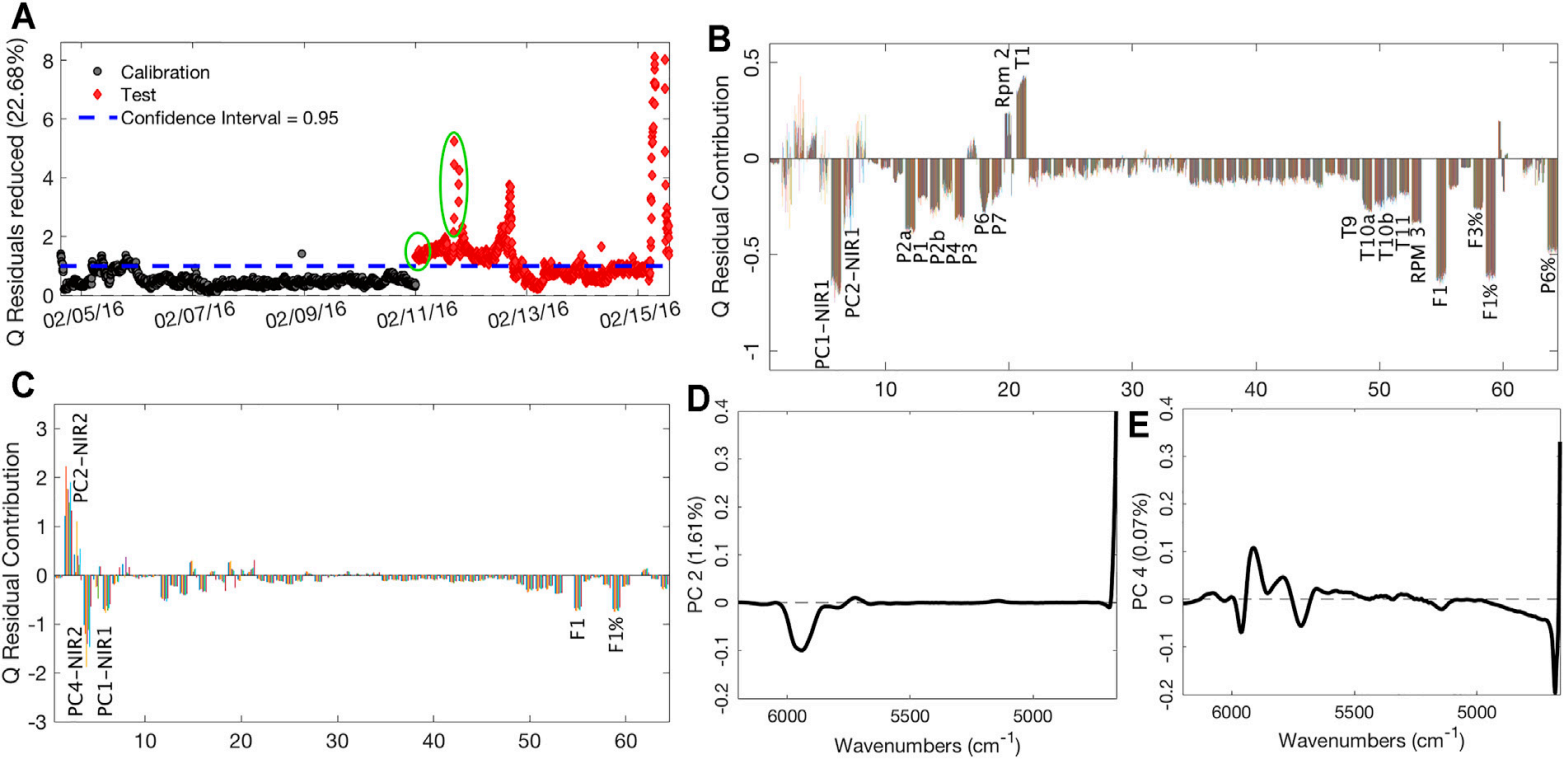
Q-based MSPC chart **(A)**; contribution plot of the first group of circled samples of **Panel A (B)**; contribution plot of the second group of circled samples of **Panel** **A** **(C)**; loadings plots on PC2 **(D)** and PC4 **(E)** of PCA performed on NIR2 dataset.

The zoom on the confidence limit area of the Q residuals MSPC chart, which describes the distance of each sample from the model space, accordingly providing information on the samples not described properly by the model, is reported in Figure 6A. It is observed that the changes in process settings, performed on February 11, caused the samples to initially fall outside the confidence interval. The contribution plot linked to these initial samples, reported in Figure 6B, shows that the PC1 scores related to the first NIR probe are the variable that mainly causes the difference with the calibration samples. In this case, it is clear and visually immediate that the process sensors concurring to explain this difference are many, suggesting plant operators to take action. The presence of the cluster of samples that present a very high Q values (Figure 6C), occurring just before the production pause, confirms the plant production problem, as highlighted by PC2-4 scores related to the second NIR probe (Figures 6D,E, respectively); thus, at this time, variations in the final product also occurred. The related loadings can be ascribed to the SAN and AN bands, suggesting, also in this case, a lower conversion of the polymer and a lower presence of AN in the final product. These observations highlight that changes in the process settings first are reflected on the intermediate product, as depicted by the NIR1 probe, and later on, the final product quality started to be nonoptimal and an intervention was operated (stop/restart), if an MSPC monitoring, like the one we analyzed retrospectively, would have been in place and a much earlier warning would have been given to the plant operators.

After the stop and the restart of the production, during which the operators worked to fix the problems, it is possible to observe a last little cluster of samples with high Q values that finally drop below the confidence limits after few hours. After that, samples remain inside the confidence interval until the moment of the formulation changes, observable by the last huge cluster of samples with high Q values.

### Predictive Models

PLS regression was used to create models capable of predicting in real time the selected quality parameters for the SAN polymer, i.e., MFI and %AN. In this part of the work, the data collected from February 15th to February 23rd, corresponding to a different formulation, was also used aiming at general predictive models. The results obtained by the four different PLS models computed as described in *Data analysis* are reported in Table 1.

**TABLE 1:** Results of PLS regression.

X block	LVs	Analysis	Calibration		Cross-validation		Prediction	
			R^2^c	RMSEC	R^2^cv	RMSECV	R^2^p	RMSEP
**NIR1**	5	**MFI (g)**	0.94	1.27	0.86	1.99	0.89	1.6
		**%AN**	0.88	0.31	0.83	0.37	0.82	0.36
**NIR2**	5	**MFI (g)**	0.94	1.23	0.84	2.12	0.86	1.92
		**%AN**	0.87	0.32	0.81	0.39	0.75	0.45
**PS**	5	**MFI (g)**	0.97	0.83	0.93	1.4	0.92	1.4
		**%AN**	0.88	0.3	0.8	0.39	0.76	0.42
**NPS**	3	**MFI (g)**	0.96	1.05	0.95	1.14	0.96	1.2
		**%AN**	0.95	0.18	0.94	0.21	0.92	0.25

For the models computed using NIR1, NIR2, and PS datasets, five latent variables (LV) were selected, whereas only three LV were considered to build the PLS model with NPS dataset. Considering the first three models, it is observable how better MFI prediction was obtained considering PS dataset, providing a prediction error of 1.4 vs. 1.6 and 1.92 g obtained using data from the first and the second NIR probes, respectively. On the other hand, %AN is slightly better predicted using the NIR1 dataset (RMSEP = 0.36%, R^2^ *p* = 0.82) rather than the other two. However, further considering the model computed using the NPS dataset, which contains both NIR and process sensors data, it is clear how it presents the best predictions for both MFI and %AN. Both internal and external validation errors, i.e., RSMECV and RMSEP, respectively, were lower than the corresponding values obtained using any of the individual datasets, whereas the related *R* ^2^ values are higher. In detail, MFI was predicted with an error of prediction equal to 1.2 g, with an R^2^ *p* = 0.96, a better prediction accuracy compared to the one obtained using the process sensors data only. This result suggests that the information NIR probes provide is important for the prediction of this quality parameter, even if the data block most significant is the one related to the process sensors. Regarding %AN, the obtained model provided an RMSEP of 0.25% and a R^2^ *p* equal to 0.92, significantly better than prediction errors and determination coefficients obtained with the other models.

## Conclusion

The current work demonstrated that the mid-level data fusion strategy, performed on the SAN polymer production process, using both NIR spectra and process sensors data, improved the quality of process control as well as the prediction ability of PLS regression models. In fact, the extraction of the features from PCA models performed on NIR data allowed to add a different and valuable kind of information to the one provided by process sensor data. T^2^- and Q-based MSPC charts computed with the NPS dataset were able to correctly detect the moments in which the process deviates from the normal operative conditions, providing at the same time information on which the sensors and/or the spectral features are linked to the problem. Furthermore, better PLS prediction of MFI and %AN parameters were obtained, in terms of RMSEP and R^2^p, using the NPS dataset rather than the ones obtained using single blocks of data.

## Data Availability

The datasets presented in this article are not readily available because of confidential agreement restrictions with the company. Requests to access the datasets should be directed to Erik.Mantovani@versalis.eni.com.
